# Evaluation of the Mechanical, Physical, and Anti-Fungal Properties of Flax Laboratory Papersheets with the Nanoparticles Treatment

**DOI:** 10.3390/ma13020363

**Published:** 2020-01-13

**Authors:** Wael A. A. Abo Elgat, Ayman S. Taha, Martin Böhm, Eva Vejmelková, Wael S. Mohamed, Yahia G. D. Fares, Mohamed Z. M. Salem

**Affiliations:** 1 Restoration department, High Institute of Tourism, Hotel Management and Restoration, Abukir, Alexandria 21526, Egypt; watsat20@yahoo.com; 2 Conservation Department, Faculty of Archaeology, Aswan University, Aswan 81528, Egypt; aymansalahtaha82@yahoo.com; 3 Department of Materials Engineering and Chemistry, Faculty of Civil Engineering, Czech Technical University in Prague, Thákurova 7, 166 29 Prague 6, Czech Republic; martin.bohm@fsv.cvut.cz (M.B.); eva.vejmelkova@fsv.cvut.cz (E.V.); 4 Polymer Department, National Research Centre, Dokki, Giza 12622, Egypt; wsabry1976@yahoo.com; 5 Laboratory and Research, Misr Edfu Pulp Writing and Printing Paper Co. (MEPPCO), Aswan 81656, Egypt; yahyagml@yahoo.com; 6 Forestry and Wood Technology Department, Faculty of Agriculture (EL-Shatby), Alexandria University, Alexandria 21526, Egypt

**Keywords:** fungal inhibition, flax papersheets, mechanical properties, nanoparticles, optical properties

## Abstract

In the present study, novel mixed additives of Chitosan or Paraloid B-72 combined with nanoparticles (NPs) of Ag, ZnO, or cellulose (NCL) were examined for their effects on the mechanical, optical, and fungal inhibition properties of the papersheets produced. The highest tensile, tear, and burst indices of the papersheets were observed for flax pulp treated with additives of Paraloid B-72 + ZnO NP (1%), Chitosan + ZnO NP (3%), and Chitosan + NCL (3%) at levels of 59.93 N·m/g, 18.45 mN·m^2^/g, and 6.47 kPa·m^2^/g, respectively. Chitosan + ZnO NP (1%) added to flax pulp showed the highest fungal mycelial inhibition (FMI) (1.85%) against *Aspergillus* *flavus*. Chitosan + Ag NP (1%) exhibited the highest FMI percentage (11.48%) when added to pulp against *A. terreus*. Pulp treated with Paraloid B-72 + Ag NP (1%) exhibited the highest activity against *Stemphylium solani* with an FMI value of 3.7%. The results indicate that the technological properties of the papersheets were enhanced with the addition of novel mixtures to the pulp.

## 1. Introduction

In the pulp and paper industry, different materials in the form of nanoparticles (NPs) are used as pulp additives or for coated paper to enhance the mechanical, physical, optical, and antimicrobial properties of the produced papersheets [1,2,3,4,5,6].

Chitosan or its derivatives are added to water-suspended paper pulp mixture with non-fibrous additives furnish [7,8] or used for coating paper [9,10,11] to obtain packaging papers with improved tensile and burst strength properties. The wet and dry tensile strength values of recycled corrugated carton pulp were improved by different dosages of high-molecular-weight Chitosan [12]. Smooth surface properties and greater resistance to humidity were observed in paper manufactured with the addition of Chitosan [13,14]. Furthermore, the addition of Chitosan improved the tensile and burst strengths of papersheets made from furnish consisting of elemental-chlorine-free (ECF) bleached Kraft pulp from spruce (70%) and totally chlorine-free (TCF) bleached Kraft pulp from birch (30%) [15].

Paper pulp with the presence of Chitosan showed good antimicrobial activities against *Staphylococcus aureus* and both fungi *Candida albicans* and *C. glabrata*, which is extremely important for paper applications in the hygiene and medical sectors [16]. Chitosan in the form of NPs was used as an antimicrobial coating in the textile industry [17]. To obtain paper with higher strength properties and lower air permeability, micro–nanochitosan additives were used [15]. Chitosan NPs as a coating material were shown to have properties of diffusion into the pores of paper fibers, resulting in increased interfibrillar bonding [11]. The molecular structure of chitosan is similar to that of cellulose, which promotes the creation of strong bonding [18]. The strength and physical properties of papersheets were effectively improved by the addition of chitosan and its derivatives as additives [19,20,21,22]. Bamboo pulp fabric treated with ZnO NPs showed potential antimicrobial properties and good ultraviolet (UV) protective properties [23].

A nanocellulose (NCL) suspension without an additional adhesive can be used to treat and consolidate weakened areas in paper [24]. NCL was used as a strengthening agent in paper, coating applications, and surface sizing due to its high tensile strength [25]. NCL mixed directly with pulp or as a filler has played an important role in paper-making due to the resulting mechanical reinforcement, improved barrier properties, optical transparency, and smoothness [26,27].

Paraloid was used for the consolidation of paper-based manuscripts, and its mixture with NPs was shown to increase or improve the mechanical and optical properties of papers [28,29], and wooden artifacts [30]. Paraloid B-72 at 2%, 3%, or 10% did not exhibit antifungal activity [30,31,32,33,34], but antifungal activity of Paraloid B-72 (10%) was observed when mixed with other substances [35].

The addition of soda–anthraquinone (AQ) in cooking liquor increases the delignification rate due to the rate of decrease in the lignin content of wood and non-wood being related to alkali consumption as the alkaline pulping process progresses. This is can be divided into three phases: (1) initial reaction, (2) bulk delignification, and (3) residual delignification [36,37,38,39]. Compared to the soda–AQ and soda method as a reference, alkaline sulphite–AQ (AS–AQ) gave the best results in terms of the yield and the mechanical and optical properties of bagasse pulp blended with bamboo pulp [40].

The aim of this research was to compare the effects of Paraloid nanocomposites in the presence of different nanoparticles with those of chitosan nanocomposites in the presence of the same nanoparticles on pulp properties and the inhibition of fungal infestation. This was achieved using novel mixtures of Chitosan or Paraloid B-72 with Ag NP, ZnO NP, and NCL at concentrations of 1% and 3%.

## 2. Materials and Methods

### 2.1. Chemicals

Chitosan powder (ACROS Organics™, Fisher Scientific, Im Heiligen Feld, Schwartz, Simmerath, Germany), 575.16–3405.35 SEK, molecular weight 100,000–300,000 KDa, Molecular formula C_56_H_103_N_9_O_39_, Molecular weight 1526.464 (g/mol), Paraloid polymer prepared by polymerization of methyl methacrylate and ethyl acrylate monomers (Aldrich, Darmstadt, Germany), ZnO NP (Aldrich, Darmstadt, Germany), Ag NP (Sigma-Aldrich, Schnelldorf, Germany), nanocellulose (NCL) (Across, Schwartz GmbH, Simmerath, Germany), acetic acid (El Gomhouria Company, Cairo, Egypt), NaOH (El Gomhouria Company, Cairo, Egypt), sodium dodecyl sulfate (SDS) (El Gomhouria Company, Cairo, Egypt), and sodium bisulphite (SBS) (Sigma-Aldrich, Schnelldorf, Germany) were used.

### 2.2. Preparation of Nanocomposites

Chitosan nanocomposite solutions were prepared by the addition of 0.03 g and 0.09 g of each of the NPs ZnO, Ag, or cellulose separately into 1% acetic acid. Each solution was mixed for about 10 min; then, 3.0 g of Chitosan powder was added and mixed vigorously and the mixture sonicated for 15 min to obtain two different concentrations (1% and 3%) of ZnO/chitosan, Ag/chitosan, and cellulose/chitosan nanocomposites. A quantity of 0.1 M NaOH was added slowly to each solution with vigorous stirring until the pH reached 6.0 and the solution was kept overnight at 60 °C [41].

The paraloid nanocomposite was prepared according to Salem et al. [42]. Briefly, a co-polymer emulsion lattice with a 50/50 composition ratio of methyl methacrylate/ethyl acrylate (MMA/EA) monomers was used to produce poly(MMA-Co-EA) [43]. It was prepared by an emulsion polymerization technique with solid content of 5% in the presence of 1% or 3% nanoparticles. The polymerization was carried out according to the following procedure: in a 250 mL three-necked flask, 1 g of emulsifier, sodium dodecyl sulfate (SDS), was dissolved in a desired amount of distilled water. The desired amount of the monomer with the selected composition ratio (50/50 MMA/EA) was added and well emulsified for 30 min at room temperature using a mechanical stirrer (500 rpm) in the presence of 1% or 3% of ZnO, Ag, or cellulose NPs, separately. Then, the mixture was heated to 80 °C [44]. Next, the redox initiation system composed of potassium persulphate (PPS) (0.27 g) and sodium bisulphite (SBS) (0.416 g) dissolved in 50 mL of distilled water was added dropwise to the reaction mixture under continuous stirring for 3 h.

It should be noted that from the literature, most of the published works which chose concentrations of NPs between 1% and 3% achieved greater modifications in the required chemical and mechanical properties of chitosan and Paraloid [29,45,46,47]. Paraloid B-72 (70 Ethyl Methacrylate (EMA)/30MA) and Chitosan were added at 4% as a constant amount when making the composite treatment.

Additionally, the polymer nanoparticles nanocomposite mixture sonicated for 15 min using pulse-echo method operating at amplitude 350 Watt and frequency of 2 MHz (central frequency of 0.7 MHz and bandwidth of 1.4 MHz). The uncertainty of the measurements is ±10 m/s using an oscilloscope (60 MHz time base oscilloscope, Philips, Eindhoven, Netherlands).

### 2.3. Morphological Analysis of the Prepared Nanocomposites

The morphological analyses of the prepared nanocomposites were performed via transmission electronic microscopy (TEM), where the TEM images were obtained using a JEM-1230 electron microscope operated at 60 kV (JEOL Ltd., Tokyo, Japan). Before taking a TEM image, the sample was diluted at least 10 times by water. A drop of well-dispersed diluted sample was placed onto a copper grid (200 mesh and covered with a carbon membrane) and dried at ambient temperature.

### 2.4. Flax Material and the Soda–Anthraxquinone Pulping Process

The flax plants grown in the North of Egypt during 2018 were used in this research with well-shaped and visually free from defects. The stems of flax plants were cut into approximately 20 mm in length, screened, and then air-dried. Preparation of the flax samples for analysis and moisture content determination were done according to T_257_ and T_208_, respectively.

Prior to pulping, 200 g oven-dried flax plant was swelled for one day, filtrated, and washed several times with hot water. The soda–anthraquinone pulping experiment was carried out using a stainless steel vessel of 3 L capacity, equipped with a rotating and heating oil bath and temperature and pressure monitor (0.7 MPa) devices. The pulping properties were active alkaline 17%, 170 °C temperature, 180 min reaction time, 0.15% anthraquinone based on the oven-dried weight (3 mL dosage from a solution of 10% anthraquinone dissolved in ethyl alcohol), and liquor ratio of 10:1 (liquid to solid). After pulping, the solid residue was defibrated, refined, and then washed with hot water and cold water to a neutral pH. The washed pulp was screened in a Valley flat screen (machine manufactured in Germany) with 0.25 mm slots.

### 2.5. Chemical Analysis

Homogenized flax samples were milled, sieved to a 60-mesh fraction, and subjected to chemical analysis. The contents (%) of holocelluloses, pentosans, benzene and alcohol extractives, lignin, and ash were measured according to the TAPPI standard methods T_249_, T_223_, T_204_, T_222_, and T_211_, respectively [48]. Furthermore, the contents of extractives soluble in cold water, hot water, and 1% NaOH were measured in accordance with the methods of T_207_, T_207_, and T_212_, respectively. The yield percentage (T_210_), kappa number (T_236_), and freeness of the pulp (T_227_) were determined for the unbleached flax pulp [48].

### 2.6. Pulp Additives

Novel mixtures of chitosan or Paraloid B-72 combined with ZnO, Ag, or cellulose nanoparticles at 1% and 3% were added based on the pulp’s oven-dried weight (Table 1). The mixture was agitated for 20 min at room temperature (25 °C). Chitosan and Paraloid B-72 as pulp additives at 4% and pulp without additives were used for comparison. All these measurements were done in triplicate.

### 2.7. Sheet Formation and Papersheet Testing

The flax pulp was beaten in a valley beater according to T_200_, then 1.6 g (oven-dry) stock was placed on a paper sheet cylinder to make standard sheets of 80 g/m^2^ with an area of 200 mm^2^ (T_205_ sp-02) [48]. For determination of the dry strength properties, the samples were conditioned at 50% ± 2% RH and 23 ± 1 °C according to T_402_ sp-98 for at least 4 h [48]. The strength properties of the papersheets were measured (T_218_ and T_220_). The produced paper sheets were tested for their tensile index (T_403_), tear index (T_414_), burst index (T_405_), double fold (T_423_) and the percentage of brightness [49] (Table 2).

### 2.8. In Vitro Inhibition of Fungal Infestation

Three fungi, *Aspergillus flavus* AFl375, *A. terreus* Ate456, and *Stemphylium solani* Ssol382, deposited in Genbank under accession numbers MH355958, MH355953, and MH355956, respectively, were used to study their growth inhibition around the produced papersheets with different additives (Table 1). Seven-day-old Potato Dextrose Agar (PDA) cultures from each fungus were prepared. Paper discs of papersheets 9 mm in diameter were put directly over the inoculated media with a disc (5 mm diameter) of each fungus in petri dishes for 14 days at 25 ± 1°C. The inhibition percentage of fungal linear growth was measured using the following formula [33]:

Mycelial growth inhibition (%) = [(A*_c_* − A*_t_*)/A*_c_*] × 100 (1)

where A*_c_* and A*_t_* represent the average diameters of the control and treatment fungal colonies, respectively.

To prevent cross contamination between samples in dishes, firstly, the paper samples were sterilized in the autoclave before being placed on the medium and secondly, the infection was done inside the laminar flow, in the presence of the UV-lamp, to complete sterilization. In addition, each dish contains only one fungus.

### 2.9. Scanning Electron Microscopy

At the end of the incubation period of the produced paper sheets with the fungi, the symptoms or inhibition of fungal infestation on the manufactured flax papersheets with different additives were examined using a Scanning Electron Microscope (SEM). The papersheet samples were coated with gold in a fine coat and examined via SEM-JEOL (JFC-1100E Ion sputtering device, model JSM- 5300, JEOL Co., Tokyo, Japan) at 8 kV.

### 2.10. Statistical Analysis

Values of the mechanical, physical, and fungal inhibition properties of the flax papersheets as affected by different pulp additives were statistically analyzed with analysis of variance (ANOVA) using the Statistical Analysis System (SAS) [50], and compared with the control treatments using Duncan’s Multiple Range Test.

## 3. Results

### 3.1. Morphological Analyses (TEM) of the Prepared Nanomaterials

**Figure 1:**
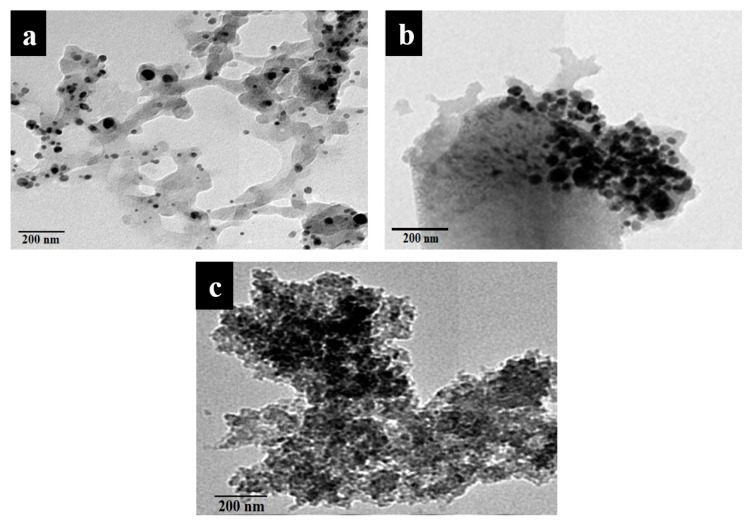
TEM photographs of the prepared nanomaterials. (**a**) Chitosan + Ag NP; (**b**) Chitosan + ZnO NP; (**c**) Chitosan + NCL.

**Figure 2:**
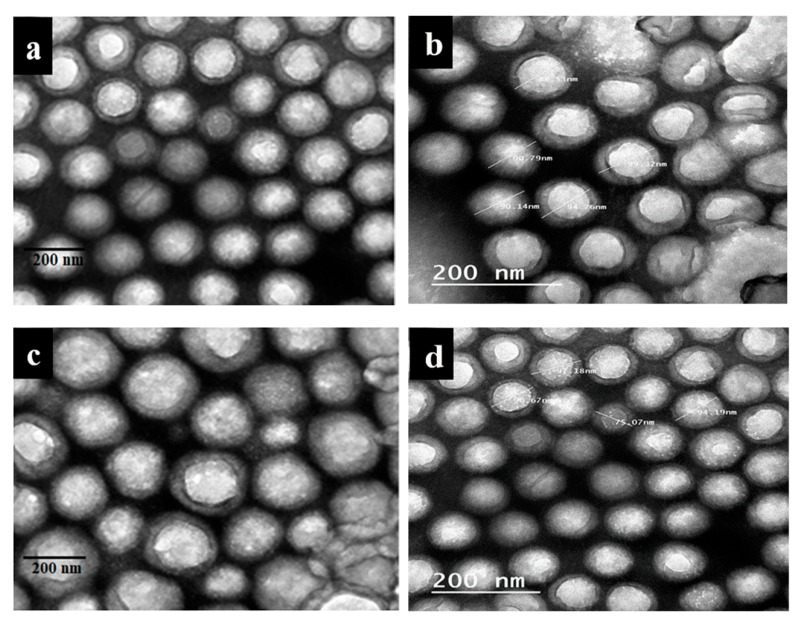

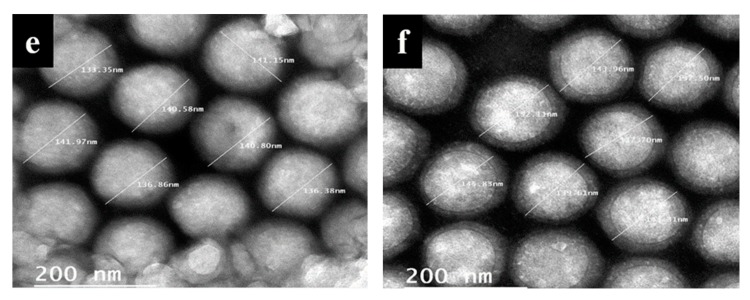
TEM photographs of the prepared nanomaterials. (**a**,**b**) Paraloid B-72 + Ag NP; (**c**,**d**) Paraloid B-72 + ZnO NP; (**e**,**f**) Paraloid B-72 + NCL.

Figure 1a–c shows TEM images of the chitosan/NP nanocomposites. NPs existed on the chitosan surface with uniform distributio n and small aggregation; the dark areas represent the NPs and bright areas represent the chitosan surface. Figure 2a–f represents TEM images of the Paraloid B-72/NP nanocomposites. The prepared nanocomposites exhibited spherical shape with particle sizes in the range of 90 nm in the case of Ag and ZnO NPs and reaching about 130 nm in the case of NCL.

TEM images represent spread of NPs over fibrous shape of chitosan in case of chitosan/NP nanocomposites, but in case of Paraloid B-72/NP nanocomposites, the NPs spread over spherical shape of Paraloid B-72. All the prepared samples are in one dimensional shape. Because both of thin film and fibrous shape are from one dimension (single dimension) nanomaterials.

### 3.2. Chemical Composition of the Flax Plant and Pulp Properties

The flax plant chemical contents of holocelluloses, pentosans, lignin, benzene:alcohol extractives, ash, solubility in cold water, solubility in hot water and solubility in 1% NaOH were 70%, 12%, 6.8%, 16%, 2.1%, 11%, and 25%, respectively. The properties of the unbleached flax pulp were found to be as follows: pulp yield (59%), kappa number (9.1), and freeness of pulp (600 CSF°).

### 3.3. Mechanical and Physical Properties of the Papersheets

**Figure 3:**
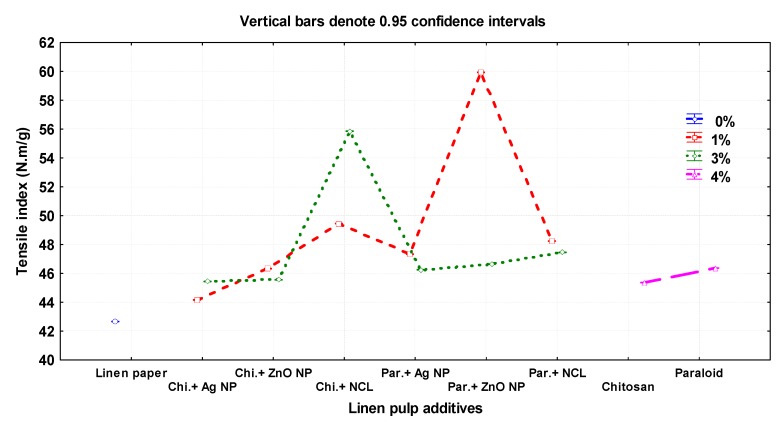

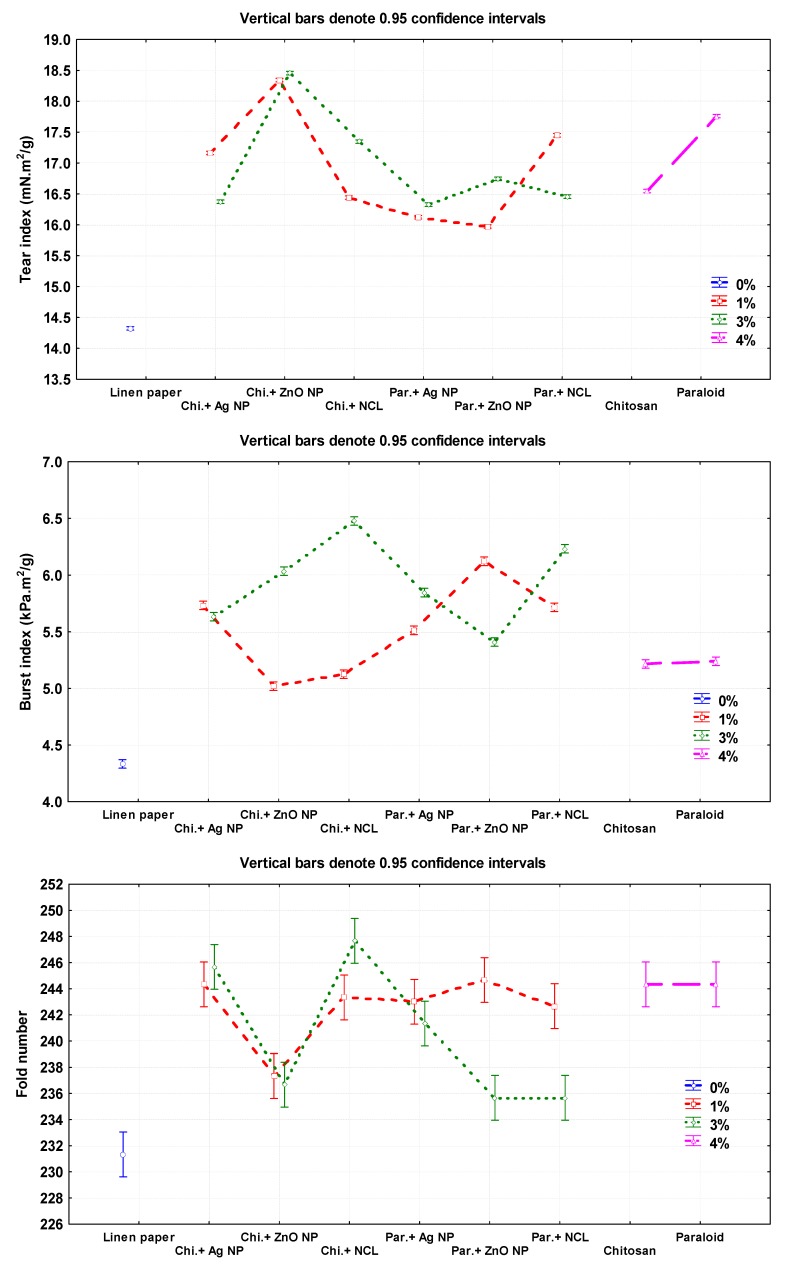

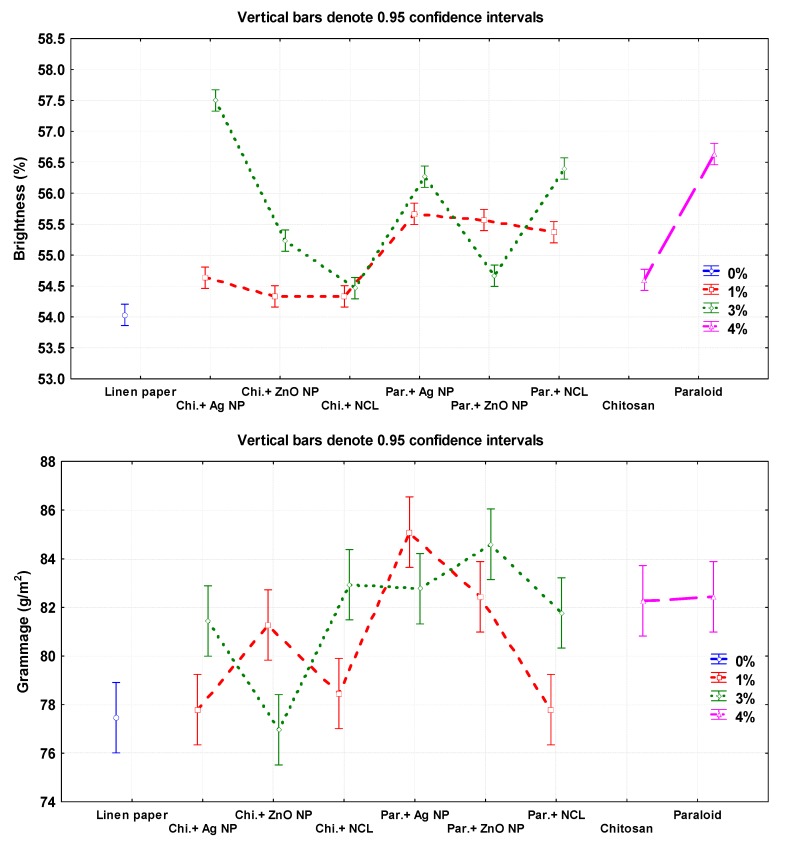
Effect of different nanomaterials on the mechanical and physical properties of the manufactured flax papersheets compared to control treatments.

Figure 3 presents the mechanical and optical properties of the manufactured flax papersheets as affected by the pulp additives and compared with the control treatments. The highest values of tensile index were observed in pulp treated with Paraloid B-72 + ZnO NP (1%), Chitosan + NCL (3%), and Chitosan + NCL (1%), with values of 59.93 ± 0.01, 55.85 ± 0.01, and 49.4 ± 0.01 N.m/g, respectively. In contrast, the lowest values were 42.66 ± 0.05, 44.16 ± 0.005, and 45.36 ± 0.01 N.m/g in the pulp without additives, Chitosan + Ag NP (1%), and Chitosan (4%), respectively.

The highest values of tear index were observed in the flax pulp treated with Chitosan + ZnO NP (3%), Chitosan + ZnO NP (1%), and Paraloid B-72 (4%), with values of 18.45 ± 0.01, 18.34 ± 0.01, and 17.76 ± 0.02 mN.m^2^/g, respectively. The lowest values were 14.32 ± 0.005 and 15.97 ± 0.02 mN·m^2^/g in pulp without additives and in that treated with Paraloid B-72 + ZnO NP (1%), respectively.

The highest burst index was achieved with flax papersheets produced from pulp treated with Chitosan + NCL (3%), Paraloid B-72 + NCL (3%), and Paraloid B72 + ZnO NP (1%), with values of 6.47 ± 0.01, 6.23 ± 0.01, and 6.12 ± 0.01 kPa·m^2^/g, respectively, compared to the control treatments of Chitosan 4% (5.21 ± 0.005 kPa·m^2^/g), Paraloid B-72 4% (5.24 ± 0.02 kPa·m^2^/g), and pulp without additives (4.33 ± 0.01 mN·m^2^/g).

The highest double fold number values were observed in flax papersheets produced with pulp additives of Chitosan + NCL (3%), Paraloid B-72 + ZnO NP (1%), Chitosan (4%), and Paraloid B-72 (4%), with values of 247.66 ± 1.52, 244.66 ± 1.52, 244.33 ± 0.57, and 244.33 ± 1.52, respectively, compared to pulp without additives (231.33 ± 0.57).

The additives Chitosan + Ag NP (3%), Paraloid B-72, and Paraloid B-72 + NCL (3%) showed the highest brightness percentages with values of 57.50% ± 0.20%, 56.63% ± 0.15%, and 56.40% ± 0.2%, respectively. The lowest values were observed in pulp without additives (54.03% ± 0.05%) and Chitosan+ZnO NP 1% (54.33% ± 0.15%).

In terms of grammage (g/m^2^), paper sheets produced from pulp with additives of Paraloid B-72 + Ag NP (1%), and Paraloid B-72 + ZnO NP (3%) exhibited the highest values of 85.09 ± 0.49 and 84.59 ± 0.49, respectively, compared to pulp without additives (77.46 ± 0.28).

The addition of Chitosan and Paraloid B-72 at 4%, as well as their combinations with Ag NPs, ZnO NPs, and NCL, led to considerable increases in the papersheets’ mechanical properties compared with the control (pulp without additives). Significant enhancements in the tensile and tear indices and in the brightness percentages were observed in pulp treated with Paraloid B-72 (4%) compared to that with Chitosan 4%, while no differences were found for burst index, fold number, and grammage.

Compared to the control treatment, pulp additives significantly enhanced (*p* < 0.05) the mechanical and physical properties of the produced flax papersheets (Table 3).

### 3.4. Biological Activity of the Flax Papersheets

**Figure 4:**
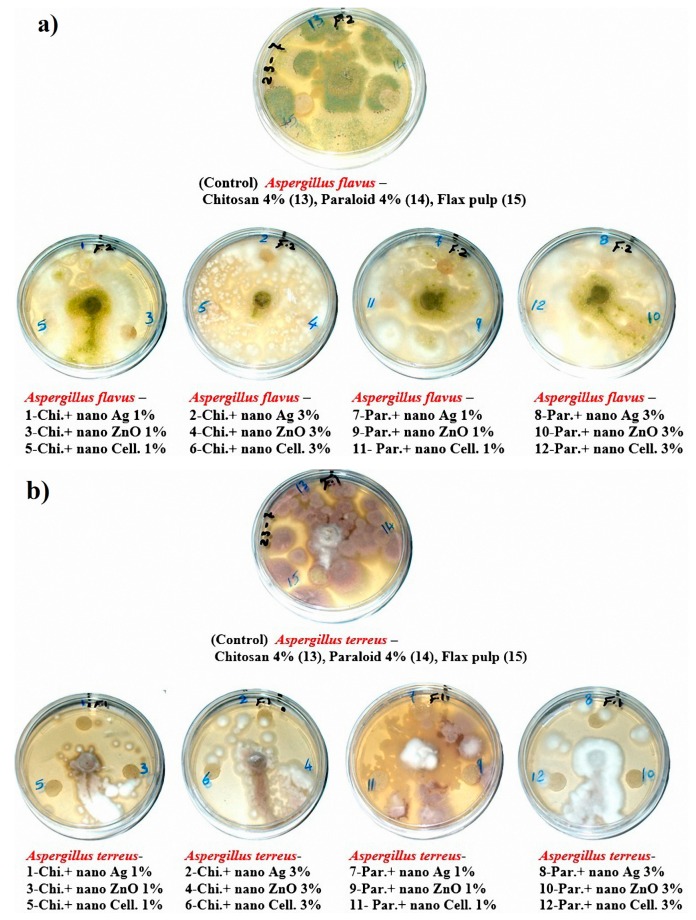

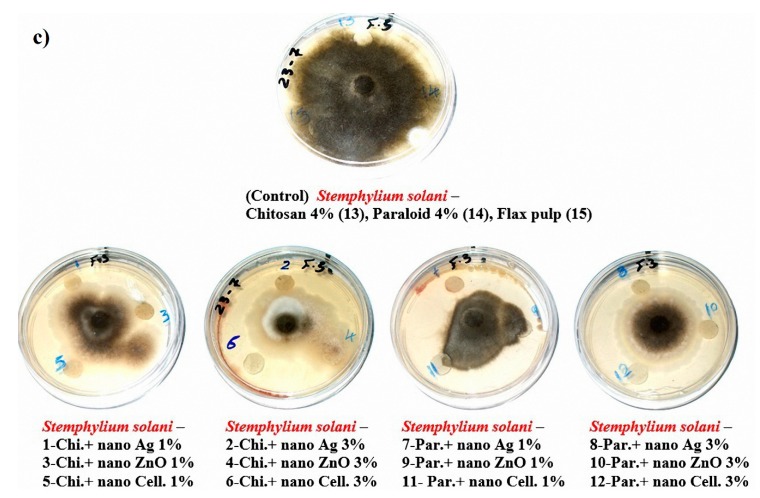
Antifungal activity of flax papersheets with different treatments against the growth of (**a**) *A. flavus*, (**b**) *A. terreus* and (**c**) *Stemphylium solani.*

The visual observations in Figure 4a–c show the degrees of fungal growth infestation of *Aspergillus flavus*, *A. terreus*, and *Stemphylium solani* colonizing the produced laboratory flax papersheets. Compared to the control treatments, *A. flavus* is clearly illustrated with intensive growth over the papersheets manufactured from the treated pulp (Figure 4a). Complete growth was found in control treatments with *A. terreus*, whereas the fungal inhibition percentage was improved for the examined papersheets made with pulp additives of Chitosan + Ag NP (1%), Chitosan + ZnO NP (1%), Chitosan + NCL (1%), Chitosan + NCL (3%), Paraloid B-72 + Ag NP (1%), Paraloid B-72 + NCL (1%), and Paraloid B-72 + NCL (3%) (Figure 4b) with different inhibition percentages of mycelial growth (Table 4). Mycilial inhibition of *S. solani* was found around the flax papersheets produced with pulp additives of Chitosan + NCL (3%) and Paraloid B-72 + Ag NP (1%) (Figure 4c).

Statistically, the antifungal activity in terms of the fungal mycelial inhibition (FMI) percentage (Table 4) showed that flax pulp with chitosan + ZnO NP (1%) additive had the highest FMI (1.85% ± 1.69%) against the growth of *A. flavus*. The highest FMI percentage values of 11.48% ± 0.64%, 10.74% ± 0.64%, 10.74% ± 0.64%, and 10.37% ± 0.64% against the growth of *A. terreus* were observed in flax papersheets produced with pulp additives of Chitosan + Ag NP (1%), Chitosan + NCL (1%), Paraloid B-72 + NCL (3%), and Chitosan + ZnO NP (1%), respectively. Pulp treated with Paraloid B-72 + Ag NP (1%), Chitosan + NCL (3%), and Chitosan + Ag NP (3%) exhibited the highest activity against *S. solani* with FMI values of 3.7% ± 0.64%, 2.22% ± 1.11%, and 2.59% ± 1.28%, respectively. On the other hand, control treatments (pulp without additives or with Chitosan (4%) or Paraloid B-72 (4%)) did not show any FMI percentages against the studied three molds.

**Figure 5:**
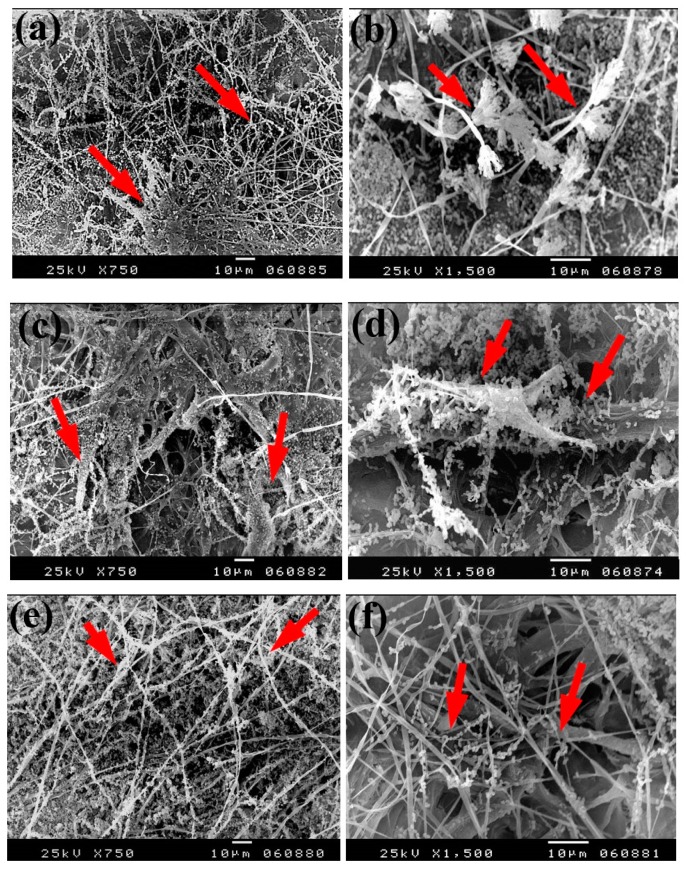

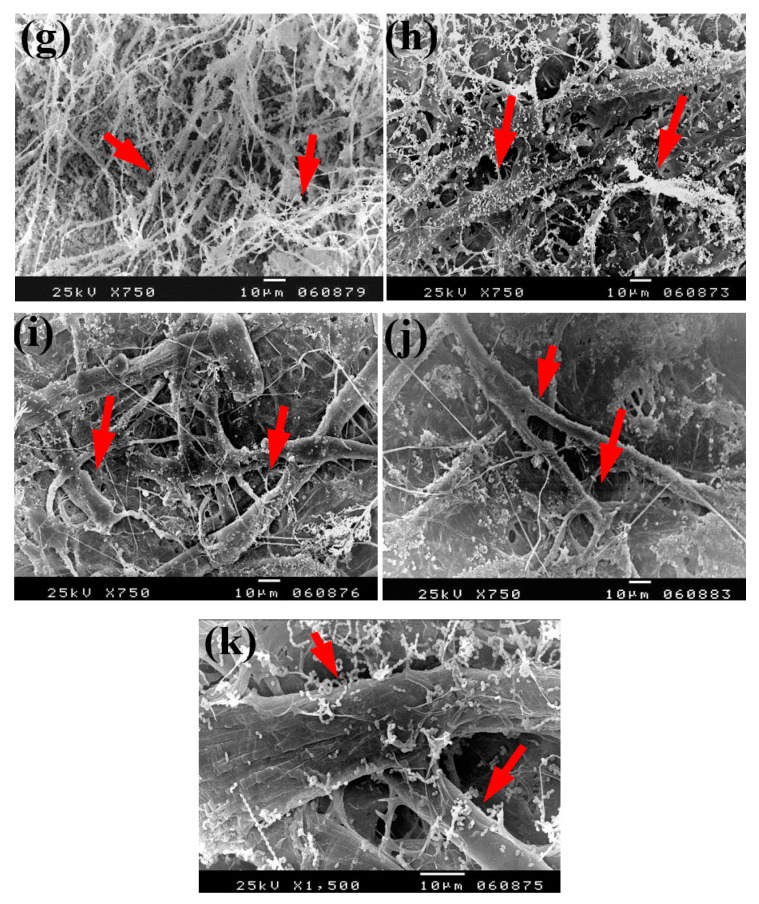
SEM images of flax papersheets manufactured with/without additives and inoculated with *A. terreus*. (**a**,**b**) Flax papersheets produced without additives; (**c**,**d**) with 4% chitosan; (**e**,**f**) with 4% Paraloid B-72; (**g**,**h**) with chitosan (4%) + ZnO NP 3%; (**i**) with chitosan 4% + Ag NP 3%; (**j**) with Paraloid B-72 4% + Ag NP 3%; (**k**) with Paraloid B-72 4% + NCL 3%. Arrows refer to growth levels of the fungus mycelium in a, b; while growth intensity varies with different concentrations of additives in c–k.

Based on the visual observations of fungal growth as well as the antifungal activities, the samples chosen for SEM measurements clearly exhibited different degrees of growth of the tested fungi. Dense and huge fungal mycelial growth of *A. terreus* (Figure 5) was clearly visible on the examined flax paper sheets produced without pulp additives (Figure 5a,b), with 4% chitosan (Figure 5c,d), and with 4% Paraloid B-72 (Figure 5e,f). Dense mycelial growth of *A. terreus* was found on the tested paper sheets manufactured with pulp additives of Chitosan + ZnO NP (3%) (Figure 5g,h). On the other hand, the fungal mycelial growth of *A. terreus* over paper samples produced with pulp additives of Chitosan + Ag NP (3%) (Figure 4i), Paraloid B-72 + Ag NP (3%) (Figure 5j), and Paraloid B-72 + NCL (3%) (Figure 5k) decreased.

**Figure 6:**
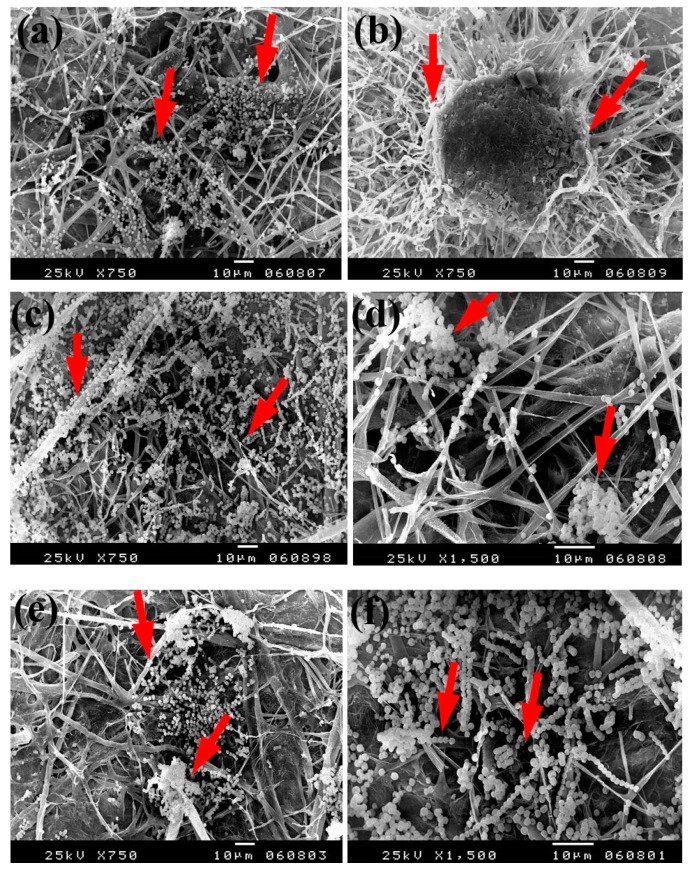

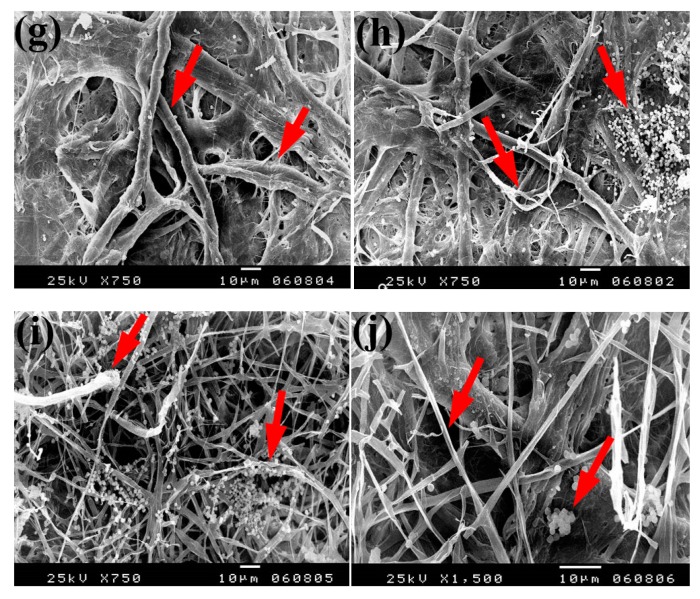
SEM images of flax papersheets produced with/without additives and inoculated with *A. flavus*. (**a**,**b**) Flax papersheets produced without additives; (**c**,**d**) with 4% chitosan; (**e**,**f**) with 4% Paraloid B-72; (**g**) with Paraloid B-72 (4%) + Ag NP 1%; (**h**) with chitosan (4%) + Ag NP 3%; (**i**) with Paraloid B-72 (4%) + ZnO NP 3%; (**j**) with Paraloid B-72 (4%) + Ag NP 3%. Arrows refer to less growth of the fungus mycelium in (**a**–**h**); while growth intensity varies with different concentrations of additives in (**i**,**j**).

The same trend was found with the growth of *A. flavus*, where huge hyphae growth was observed over the papersheets produced without pulp additives (Figure 6a,b), with 4% chitosan (Figure 6c,d), and with 4% Paraloid B-72 (Figure 6e,f). Flax papersheets produced with pulp additives of Paraloid B-72 + Ag NP 1% (Figure 6g) and Chitosan + Ag NP 3% (Figure 6h) showed reasonable decreases in the hyphae growth of *A. flavus*, while dense growth was observed in pulp treated with Paraloid B-72 + ZnO NP 3% (Figure 6i) and Paraloid B-72 + Ag NP 3% (Figure 6j).

**Figure 7:**
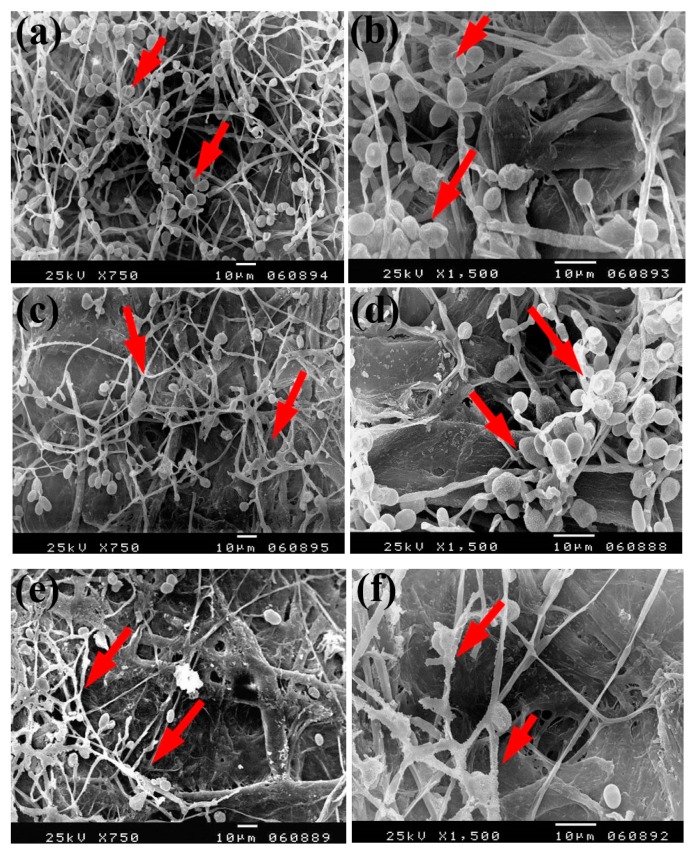

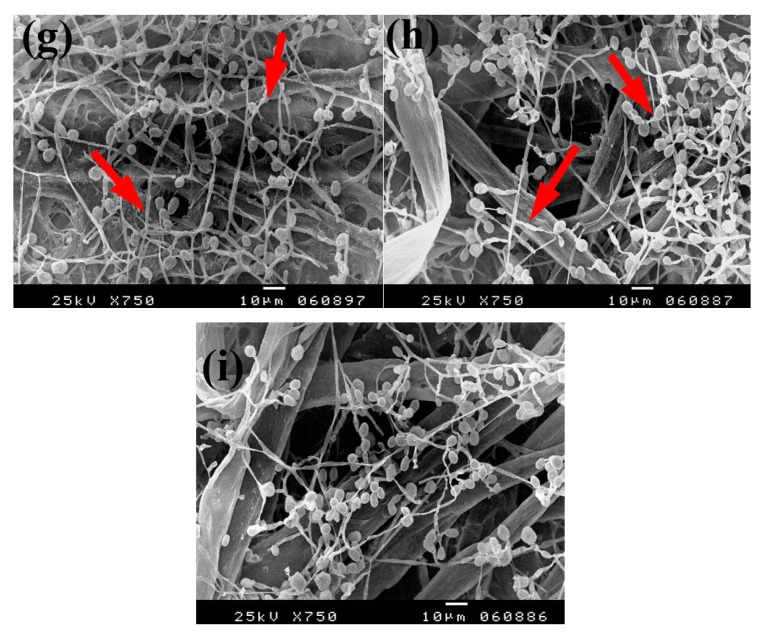
SEM images of flax papersheets manufactured with the addition of different additives and inoculated with *Stemphylium solani*. (**a**,**b**) Flax papersheets without additives; (**c**,**d**) with 4% chitosan; (**e**,**f**) with 4% Paraloid B72; (**g**) with chitosan 4% + ZnO NP 3%; (**h**) with Paraloid B72 4% + ZnO NP 1%; (**i**) with chitosan 4% + NCL 1%. Arrows refer to the growth of hyphae.

Dense growth of *S. solani* was observed in flax paper sheets manufactured without pulp additives (Figure 7a,b), with 4% chitosan (Figure 7c,d), and with 4% Paraloid B-72 (Figure 7e,f). Furthermore, dense growth was observed in pulp treated with chitosan + ZnO NP 3% (Figure 7g), Paraloid B-72 + ZnO NP 1% (Figure 7h), and chitosan 4% + NCL 1% (Figure 7i).

Control treatments did not exhibit any antifungal activities against the growth of *A. flavus*, *A. terreus*, or *S. solani*. This indicates that the antifungal properties of flax pulp were significantly improved when the flax pulp was treated with nanocomposite additions of chitosan 4% or Paraloid B-72 4% combined with Ag NP or NCL at 3%.

## 4. Discussion

The TEM images showed the prepared of nanomaterials, where the aggregation of NPs on the chitosan surface increased from Ag to ZnO, and high aggregation was observed in the case of NCL, which could be related to the increasing particle size of NCL relative to Ag and ZnO NPs [51]. Additionally, the NPs were uniformly distributed on the surface of the polymer matrix, which confirmed the successful preparation of Paraloid B-72 nanocomposite with Ag NPs, ZnO NPs, and NCL [29].

According to the chemical analysis of flax plant, the lignin content was much lower than that in hardwood (25–30%), but the ash content was higher than values obtained from hardwood species (0.2–1.5%) [52,53].

Pulp additives were significantly affected the mechanical properties of the manufactured flax papersheets. Tensile index values are lower than those values reported from soda–AQ pulping of bagasse (77.8–73.8 N·m/g) [40]. Our results are in agreement with those by Jahan et al. [54] who found that the average value of the tensile index of papersheets produced from soda–AQ pulping of *Acacia auriculiforms* reached 45.1 N·m/g. The present values of tear index are higher than those reported from papersheets produced from soda–AQ pulping of bagasse, with values ranged from 5.7 to 6.0 mN·m^2^/g [40] and from *A. auriculiformis* (3.7 to 6.7 mN·m^2^/g) [54]. Comparative to other lignocellulosic plants, burst index values are higher than those reported in the literature (kPa·m^2^*/*g) for stems of *Stipa tenacissima* (1.3), bamboo (2.02), giant reed (0.5), miscanthus (1.23), reed canary (4), switch grass (5.3), Napier grass (4.98), and bagasse (4.8–4.9) [40,55,56,57,58,59,60,61].

In the present study, the treatments of Chitosan + NCL (1% or 3%) and Paraloid B72+ZnO NP (1%) increased the tensile strength of the paper sheets. These results are in agreement with Vikele et al. [15] who found that micro–nanochitosan increased the tensile index and concluded that micro–nanoparticles fill the submicroscopic voids of the porous paper structure and create additional bonds. NCL, with its high surface area and flexibility, increases the strength of the network [62,63] by increasing the number of hydrogen bonds between each fibril and fibers [64]. Pulp additives with 6% (dry weight) NCL showed a resulting increase in the tensile strength of the produced 60 g/m^2^ papersheets by 26% to 30% [65], and the same trend was found by Bilodeau and Bousfield [66], Hamann [67], and Madani et al. [68]. NCLs enhanced the fiber–fiber bond strength; subsequently, a strong reinforcing effect in paper and board products occurred [69].

Results of the SEM are in agreement with previous works wherein at 2% or 10% Paraloid B-72, no increases in the resistance of beech and spruce wood were observed against *Coniophora puteana* and *Gloeophyllum trabeum* [31], and weak activity was observed against *Poria vaillantii* [32]. In addition, some fungi are able to grow on Paraloid B-72 [70]. On the other hand, a combination of Paraloid B-72 (10%) with Pentachlorophenol 2% showed antifungal activity against *A. flavus* [35]. Wood treated with Paraloid B-72 (2% or 3%) showed huge mycelial growth of *Trichoderma harzianum* [33], *Alternaria tenuissima*, and *Fusarium culmorum* [34].

Previously, Chitosan in its free polymer form was proven to exhibit potential antifungal activity against *A. niger*, *A. alternata*, *Rhizopus oryzae*, *Phomopsis asparagi*, *R. stolonifera*, *Botrytis cinerea*, and *F. oxysporum* [71,72,73,74]. In the present study, pulp with Chitosan applied as an additive showed intense growth of fungi. However, other pulp additives that showed superior or stronger results for preventing fungal growth can be seen in Table 4 and Figure 4a–c. *Chaetomium globosum* growth significantly affects the dry mass as well as the tensile elastic modulus of some tested natural fiber mats and composites including non-woven flax fibers [75].

Overall, the enhancing effects of additives on the technological properties are much greater than the antifungal activities of the produced flax papersheets.

## 5. Conclusions

In this study, additives were used to enhance the mechanical, optical, and antifungal properties of paper sheets manufactured from flax pulp. Remarkable enhancement in the tensile index was found in pulp treated with Paraloid B-72 + ZnO NP 1% and Chitosan + NCL (1% or 3%) compared to control treatments (pulp without additives, with chitosan 4%, or with Paraloid B-72). Addition of Chitosan + ZnO NP (1% or 3%) and Paraloid B-72 4% increased the Tear index values. Furthermore, the burst index values of the paper sheets were enhanced with the addition of Chitosan + NCL (3%), Paraloid + NCL (3%), and Paraloid + ZnO NP (1%), while the double fold number was improved with the addition of Chitosan + NCL (3%), Paraloid B-72 + ZnO NP (1%), Chitosan 4%, or Paraloid B-72 4%. Pulp additives significantly affected the optical properties of the produced papersheets. The novel combination treatments can be considered to produce antifungal papersheets when compared to the huge growth of *Aspergillus flavus*, *A. terreus*, and *Stemphylium solani* that was observed over papersheets produced with pulp with additives of Chitosan and paraloid B-72 at 4% as well as pulp without additives.

## Figures and Tables

**Table 1:** Different pretreatments used for flax pulp.

	Treatment
1	Chitosan + Ag NP 1%
2	Chitosan + Ag NP 3%
3	Chitosan + ZnO NP 1%
4	Chitosan + ZnO NP 3%
5	Chitosan + NCL 1%
6	Chitosan + NCL 3%
7	Paraloid + Ag NP 1%
8	Paraloid + Ag NP 3%
9	Paraloid + ZnO NP 1%
10	Paraloid + ZnO NP 3%
11	Paraloid + NCL 1%
12	Paraloid + NCL 3%
13	Chitosan 4%
14	Paraloid B-72 4%
15	Pulp without additives

**Table 2:** Description test machines and their specifications.

Test Machine	Specifications
Tensile Tester	Model: Adamel Lhomargy, model No. 596420, DY-30; Maximum load cell: 100 N display in Newton or KN; Digital display: 41/2 digits; Accuracy: 0.1%; Speed range: 0.01 to 999 mm/min, automatic return sped.
Tear Tester	Model: FRANK-PTI GMBH, Elmendorf tear tester, digital, Mod. 53984, Sr. 40551, Germany; Fully automatic model Available pendulums: 0–8000 mN; Compressed air 4–6 bar.
Burst Tester	Model: Tecnolab Company, model No. BS 20 E/SN. 160.08, Italy; Capacity 1999 kPa; Sensitivity 1 kPa; Accuracy ±0.5% kPa; Pump flow rate 95 ± 5 mL/min; Circular clamp diameter 65 mm; Diaphragm diameter 30.5 mm; Adjustable clamp pressure 0–90 psi; Air supply 6 bar max (90 psi).
Twin Folding Tester	Model: KÖGEL LEIPZIG, DFP 6-60; Standard tension of 9.81 N; Sample length: 100 mm; Sample width: 15 mm; Speed: 115 ± 10 strokes/min
Color Touch Model ISO	Model: Technidyne Corporation, New Albany Indiana USA, Model NO. CTH- ISO, Serial NO. CTH A 2054; Technidyne Corporation.

**Table 3:** ANOVA analysis of the effect of different treatments on the mechanical and physical properties of papersheets made from the flax plants.

SOV	DF	Sum of Squares	Mean Square	F Value	Pr > F
Tensile index (N·m/g)					
Additives (A)	6	383.552	63.925	198389	<0.0001
Concentrations (B)	1	17.056	17.056	52935.2	<0.0001
A × B	5	315.438	63.087	195789	<0.0001
Error	30	0.0096	0.00032		
Corrected Total	44	837.958			
Tear index (mN·m^2^/g)					
A	6	20.284	3.3807	5828.86	<0.0001
B	1	0.011	0.011	19.01	<0.0001
A × B	5	4.608	0.922	1589.27	<0.0001
Error	30	0.0174	0.0006		
Corrected Total	44	44.703			
Burst index (kPa·m^2^/g)					
A	6	0.668	0.112	110.02	<0.0001
B	1	1.444	1.444	1425.00	<0.0001
A × B	5	4.185	0.837	826.09	<0.0001
Error	30	0.0304	0.001		
Corrected Total	44	12.645			
Fold number					
A	6	337	56.166	26.61	<0.0001
B	1	40.111	40.111	19.00	<0.0001
A × B	5	190.55	38.111	18.05	<0.0001
Error	30	63.333	2.11		
Corrected Total	44	985.20			
Brightness (%)					
A	6	20.936	3.489	163.89	<0.0001
B	1	5.359	5.359	251.71	<0.0001
A × B	5	11.555	2.3111	108.55	<0.0001
Error	30	0.638	0.0212		
Corrected Total	44	44.07			
Grammage (gm/m^2^)					
A	6	131.195	21.866	14.50	<0.0001
B	1	14.554	14.554	9.65	0.0041
A × B	5	102.238	20.447	13.56	<0.0001
Error	30	45.23	1.5077		
Corrected Total	44	342.18			

SOV: source of variance; DF: degrees of freedom.

**Table 4:** Mycelia percentage inhibited of *A. flavus*, *A. terreus* and *S. solani* by pulp treated with different nanoparticle materials at different concentrations.

Pulp Additives	Concentration (%)	Inhibition of Mycelial Growth (%)		
		*Aspergillus flavus*	*Aspergillus terreus*	*Stemphylium solani*
Chitosan + Ag NP	1	1.48 ± 1.28	11.48 ± 0.64	0.74 ± 0.64
	3	1.11 ± 1.11	8.51 ± 2.79	2.59 ± 1.28
Chitosan + ZnO NP	1	1.85 ± 1.69	10.37 ± 0.64	1.85 ± 1.69
	3	1.48 ± 1.28	2.59 ± 1.28	0.74 ± 0.64
Chitosan + NCL	1	1.11 ± 1.11	10.74 ± 0.64	0.37 ± 0.64
	3	0.74 ± 0.64	5.92 ± 0.64	2.22 ± 1.11
Paraloid B-72 + Ag NP	1	0.37 ± 0.64	8.88 ± 1.11	3.7 ± 0.64
	3	0.74 ± 1.28	9.62 ± 0.64	1.48 ± 1.28
Paraloid B-72 + ZnO NP	1	0.37 ± 0.64	8.14 ± 1.69	0.37 ± 0.64
	3	0.00	8.14 ± 1.28	1.85 ± 0.64
Paraloid B-72 + NCL	1	0.37 ± 0.64	8.14 ± 1.28	0.37 ± 0.64
	3	0.00	10.74 ± 0.64	0.74 ± 1.28
Without additives	0	0.00	0.00	0.00
Chitosan	4	0.00	0.00	0.00
Paraloid B-72	4	0.00	0.00	0.00
*p*-value		**	**	**

Notes: Values (**) are presented as mean ± SD.
